# Expression of ezrin protein and phosphorylated ezrin in pelvic endometriotic lesions

**DOI:** 10.1016/j.clinsp.2022.100074

**Published:** 2022-07-03

**Authors:** Alessandra Peloggia, Marina Paula Andres, Mauricio Simões Abrão

**Affiliations:** a Centro de Pesquisa em Saúde Reprodutiva de Campinas (CEMICAMP), Campinas, SP, Brazil; b Departamento de Obstetrícia e Ginecologia, Hospital das Clínicas, Faculdade de Medicina, Universidade de São Paulo (HCFMUSP), São Paulo, SP, Brazil; c Gynecologic Division, BP ‒ A Beneficencia Portuguesa de São Paulo, São Paulo, SP, Brazil

**Keywords:** Ezrin, Phosphorylated Ezrin, Endometriosis, Immunology

## Abstract

**Objective:**

To evaluate the expression of Ezrin and Phosphorylated Ezrin (Phospho-Ezrin) in endometriosis lesions and its relation to the menstrual cycle phase, stage of endometriosis, histological classification, and clinical symptoms.

**Material and methods:**

The authors conducted a retrospective study, with endometriotic lesions collected from women with endometriosis (n = 57) who underwent laparoscopy from 2017 to 2018. The expression of Ezrin and Phosphorylated Ezrin proteins was analyzed by immunohistochemistry.

**Results:**

All the endometriotic lesions contained immunostaining for Ezrin in the glands. Phosphorylated Ezrin was expressed in the stroma of all endometriotic lesions. There was no difference in the Ezrin and Phosphorylated Ezrin's expression in the retrocervical, ovarian, superficial, and intestinal lesions in the same patient. Dysmenorrhea, dyspareunia, acyclic pain, infertility, and dysuria were similar in the three groups of Ezrin staining. There was an inversely proportional relationship between severe dyschezia and Ezrin's intensity, being 66.7% of Ezrin 1 (weak intensity), 36.7 Ezrin 2 (moderate intensity), and 10.0% of Ezrin 3 (p = 0.013). Regarding Phospho-Ezrin there wasn't a significant difference between all the analyzed variables. Histological classification and menstrual cycle phase had also no significant difference between Ezrin and Phospho-Ezrin immunostaining.

**Conclusion:**

Ezrin protein and Phospho-Ezrin can be considered important markers to elucidate the mechanisms related to migration and attachment of endometriotic lesions. It is still unclear if Ezrin and Phospho-Ezrin are a cause or consequence of endometriosis. Further studies comparing different types of lesions and eutopic endometrium are necessary to elucidate the role of these proteins in the pathogenesis of endometriosis.

## Highlights

• The implantation of endometrial cells in the pelvic cavity has been related to some factors such as a receptive environment that allows the implantation and proliferation of these cells.
• Several studies have shown the participation of the Ezrin protein in the process of invasion of malignant cells.
• The expression of Ezrin and its activated form was observed in endometriotic lesions providing great evidence that these proteins can play an important role in the migration and attachment of endometriotic lesions.

## Introduction

Endometriosis is a chronic inflammatory disease, estrogen-dependent, and defined as the presence of endometrial glands and stroma outside the uterus, affecting 10%‒15% of reproductive-aged women.^1^^,^^2^

Endometriosis is commonly associated with infertility, chronic pelvic pain, dysmenorrhea, and deep dyspareunia.^3^ Intestinal and urinary symptoms can also occur depending on the lesions’ location.^4^ Deep endometriosis is differentiated from peritoneal and ovarian endometriosis by the infiltration deeper than 5 mm of the ectopic endometrium into the peritoneum.^5^

The pathogenesis of endometriosis remains undefined, but the retrograde menstruation theory^6^ is the most widely accepted, where endometrial cells reach the abdominal cavity, through the fallopian tubes during menses. Besides this, there are a great number of studies reporting that cytokines, matrix metalloproteinases proteins, and growth factors are also involved in endometriosis development.^1^

The essential step for ectopic endometrial cells to implant and grow is the cell-to-cell or cell-to-extracellular matrix (ECM) interaction with the peritoneal surface.^7^ These interactions are mediated by the integrin family (adhesion molecules) of cell surface receptors related to linker proteins called ERM (Ezrin, Radixin, Moesin).^8^

Ezrin is a structural protein that organizes receptors and commands their transduction, localized beneath the plasma membrane of cellular protrusions and at cell-to-cell junctions of the cytoskeleton.^7^^,^^9^ Ezrin also interacts with cell surfaces adhesion molecules such as CD44 and ICAM-1, playing an important role in the regulation of migration, morphology, and cell adhesion.^8^

Ezrin is normally an inactive protein due to intramolecular interaction between its N and C terminal domains. Phosphorylation can occur at different ways to activate the Ezrin. One way is the phosphorylation at C-terminal threonine residue, which causes dissociation between these terminals, playing a crucial role in modulating the function of the ERM proteins., specifically in the formation of cell surface projections.^9^ Thereby, the phosphorylated Ezrin (Phospho-Ezrin) becomes the activated form of Ezrin and may participate in cell adhesion and invasion.

Some studies previously evaluated the relation between Ezrin and cancer^8^^,^^10^, ^11^, ^12^ and also the relationship between these proteins and endometriosis. Ornek et al.^13^ reported an increase of expression of Ezrin and Phosphorylated Ezrin in the eutopic and ectopic endometrium of women with endometriosis when compared to the endometrium of women without endometriosis and in addition, the stromal cells of women with endometriosis had more invasive characteristics and high immunostaining for Ezrin when compared to the control group.

Since these proteins play a role in cell attachment and invasion, and in order to investigate their role in the pathogenesis of endometriosis, the aim of the present study is to evaluate the expression of Ezrin and Phosphorylated Ezrin in endometriotic lesions and their relation with the menstrual cycle phase, stage of endometriosis, histological classification, and clinical symptoms.

## Material and methods

The authors conducted a retrospective study, with endometriotic lesions collected from women with endometriosis who underwent laparoscopy from 2017 to 2018 at Hospital BP ‒ A Beneficência Portuguesa de São Paulo and Hospital das Clínicas da FMUSP, a tertiary referral center in São Paulo, Brazil. The surgical indication was based on clinical symptoms, failure in hormonal treatment, and findings of suspected endometriosis on transvaginal ultrasound with bowel preparation or Magnetic Resonance imaging. The study was approved the by Hospital BP Ethical Committee (n° 0386/11).).

The study population included consecutive women aged 18‒45 years with histological confirmation of endometriosis, with regular menstrual cycles (interval between cycles ranging from 26‒34 days), without hormonal treatment for three months prior to the laparoscopy. Women with autoimmune diseases, irregular menstrual cycles, and any gynecological infection disease were excluded from the study.

The symptoms evaluated were dysmenorrhea, deep dyspareunia, infertility, non-cyclic pelvic pain, cyclic dyschezia, and cyclic dysuria. Pain symptoms were evaluated using the Visual Analogue Scale (VAS) ranging from 0 (no pain) to 10 (incapacitating pain). Infertility was defined as the absence of pregnancy after one year of unprotected sexual intercourse. Patients were classified according to the American Society for Reproductive Medicine (ASRM, 1996) in four stages (I‒IV).

### Immunohistochemistry

The expression of both Ezrin and the Phosphorylated Ezrin was evaluated through immunohistochemistry. The samples collected during the laparoscopy were fixed in 10% formalin, buffered for 18‒24 hours, and then underwent routine histological processing to paraffin blocks. Sections measuring 5 μm were cut in a microtome and placed on glass slides.

For selection and identification of areas of interest, a microscopic analysis of the stained Hematoxylin and Eosin (HE) was performed using a marker pen. At this time, the authors considered the epithelial/estromal component and the lesion size.

Immunohistochemistry was performed according to the procedures that have been described previously.^13^ Tissue samples were fixed in ethanol 100% and dehydrated in alcoholic solutions in decreasing concentrations of ethanol. After that, to unmask antigens, samples were incubated for 5 minutes in a microwave oven at 750W, followed by cooling at room temperature for 20 minutes, placed in citrate buffer (pH 6), and washed in phosphate-buffered saline (PBS; pH 7.4). In order to remove endogenous peroxidase activity, sections were placed in 3% hydrogen peroxide for 30 minutes and then washed three times with PBS. Posteriorly slides were incubated in a humidified chamber with blocking serum for 10 minutes at 20°‒25°C.

The following antibodies were used at a dilution of 1:500: Monoclonal mouse anti-human ezrin IgG1 isotype (1:600; Clone 3C12, Sigma-Aldrich, Inc.) and Rabbit anti-human phospho-ezrin antibody (1:800; TYR-353, Cell Signaling Technology, Beverly, MA, USA). The slides were incubated with the primary antibodies for one hour at 20°‒25°C, and washed three times for 5 minutes with PBS. Then the secondary antibodies (dilution 1:500, biotinylated horse anti-mouse or anti-rabbit antibody (1.5 mg/mL; Vector Laboratories, Burlingame, CA, USA) were added and incubated for 45 minutes at 20°‒25°C. The antigen-antibody complex was detected by using an avidin-biotin-peroxidase kit (Lab Vision, Värmdö, Sweden). Diaminobenzidine (3,3-diaminobenzidine tetrahydrochloride dehydrate; LabVision, Värmdö Sweden) was used as the chromogen, followed by counterstaining with Mayer's hematoxylin solution (Merck, Darmstadt, Germany). The internal quality controls procedures were performed by carrying out negative controls, where the primary antibodies were replaced by appropriate non-immune IgG isotypes.

### Analysis of immunostaining

Staining was assessed with the use of optical microscopy (× 400) and the intensity of immunostaining was semi-quantitatively evaluated and the positivity of stained cells was grouped as follows: 0 (negative- no staining); 1 (weak, but detectable); 2 (moderate); 3 (intense).

### Statistical analysis

For parametric data, when comparing three or more independent groups with similar variances according to Bartlet's Test, the authors used the Analysis of variance. Kruskal-Wallis test with Bonferroni correction was used for non-parametric data when comparing three independent groups.

Categorical variables were analyzed as absolute numbers (n) and frequencies (%) and compared by Pearson's Chi-Square test or Fisher's exact test. Value of p ≤ 0.05 was considered statistically significant for this study.

## Results

A total of 129 patients were eligible for the study, of whom, 60 were excluded for being under hormonal treatment, 3 for being older than 45 years, and 9 for having irregular menstrual cycles, which resulted in 57 women included. The clinical characteristics of the selected women are shown in Table 1.

**Table 1:** Menstrual cycle phase, type of endometriotic lesion, stage of the disease, and Ezrin immunostaining.

Characteristics	Ezrin			p
	1	2	3	
Age (years)	38.0±4.2	36.2±6.0	35.4±4.4	0.540^a^
BMI (kg/m^2^)	22.1±1.7	24.1±6.0	23.4±3.0	0.522^b^
Phase of the menstrual cycle				0.282^c^
Proliferative	2 (8.7)	10 (43.5)	11 (47.8)	
Secretory	5 (14.7)	20 (58.8)	9 (26.5)	
ASRM Stage				0.921^c^
I	0 (0)	2 (6.7)	2 (10.0)	
II	2 (28.6)	10 (33.3)	8 (40.0)	
III	2 (28.6)	6 (20.0)	2 (10.0)	
IV	3 (42.9)	12 (40.0)	8 (40.0)	
Dysmenorrhea (VAS)				0.556^c^
< 7	1 (16.7)	10 (33.3)	4 (20.0)	
≥ 7	5 (83.3)	20 (66.7)	16 (80.0)	
Dyspareunia (VAS)				0.703^c^
< 7	5 (83.3)	21 (70.0)	13 (65.0)	
≥ 7	1 (16.7)	9 (30.0)	7 (35.0)	
Acyclic pain (VAS)				0.195^c^
< 7	4 (66.0)	24 (80.0)	11 (55.0)	
≥ 7	2 (33.3)	6 (20.0)	9 (45.0)	
Cyclic Dyschezia (VAS)				**0.013**^c^
< 7	2 (33.3)	19 (63.3)	18 (90.0)	
≥ 7	4 (66.7)	11 (36.7)	2 (10.0)	
Cyclic dysuria (VAS)				0.088^c^
< 7	5 (83.3)	30 (100)	19 (95.0)	
≥ 7	1 (16.7)	0 (0)	1 (5.0)	
Infertility				0.240^c^
No	0 (0)	7 (29.2)	2 (11.8)	
Yes	6 (100)	17 (70.8)	15 (88.2)	

Data expressed as mean±standard deviation or n (%); BMI, Body Mass Index; ASRM, American Society for Reproductive Medicine, 1996; VAS, Visual Analogic Scale from 0 to 10.

a ANOVA.

b Kruskal-Wallis Test.

c Fischer's Exact Test.

**Figure 1:**
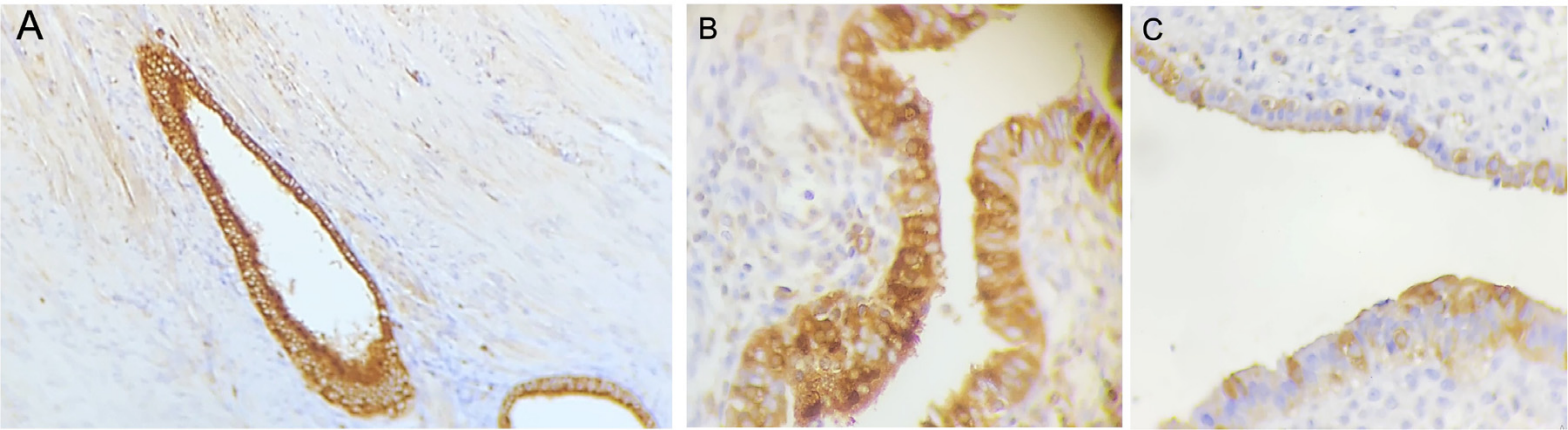
Ezrin immunohistochemistry in the glands of endometriosis lesions. (A) Score 3 (strong) in well-differentiated glandular endometriosis histological type (20 ×). (B) Score 2 (moderate) in well-differentiated glandular endometriosis histological type (40 ×). (C) Score 1 (weak) in indifferent endometriosis histological type (40 ×).

**Figure 2:**
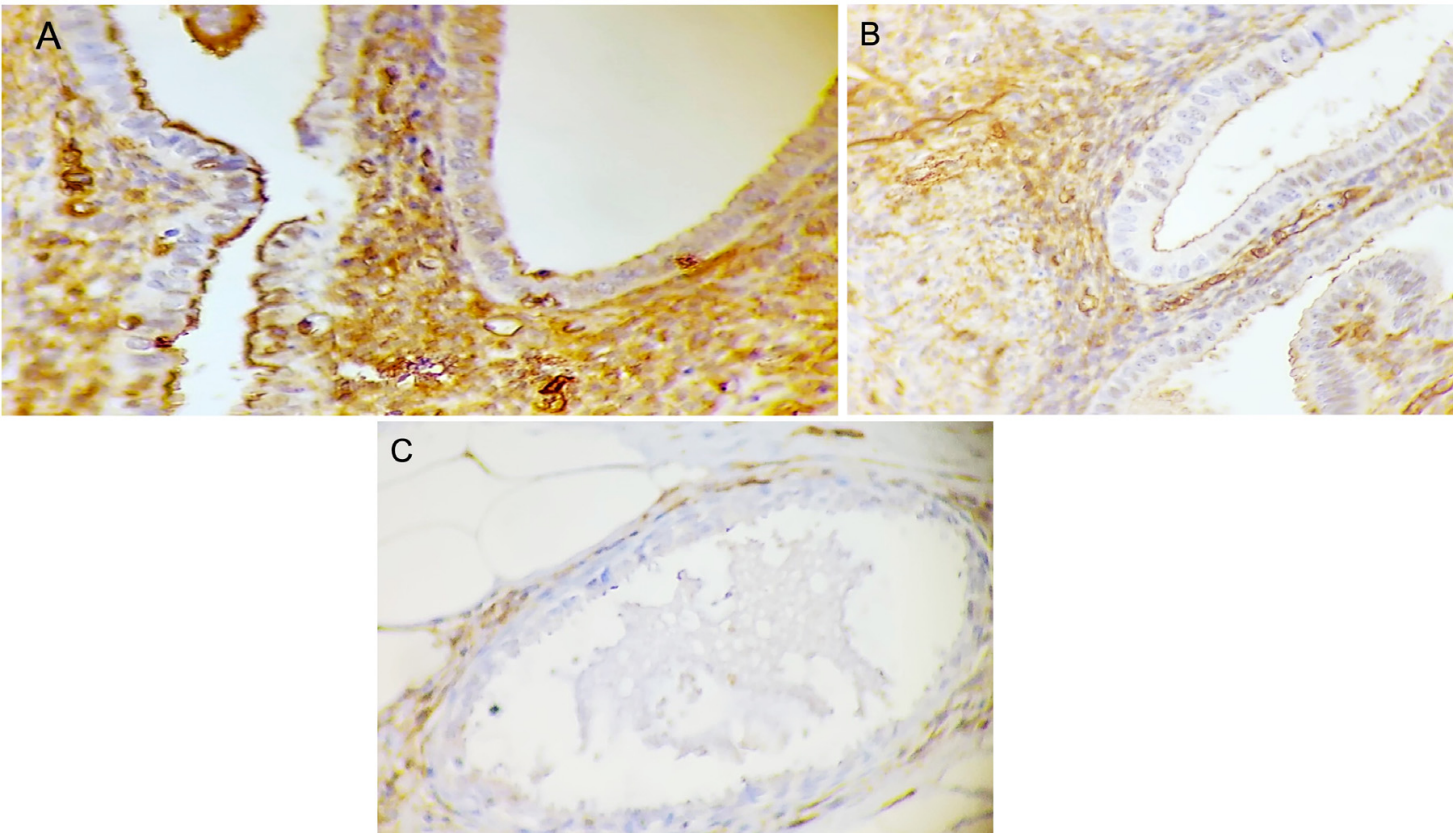
Phosphorylated Ezrin immunohistochemistry in the stroma of endometriosis lesions. (A) Score 3 (strong) in well-differentiated glandular endometriosis histological type (40 ×). (B) Score 2 (moderate) in well-differentiated glandular endometriosis histological type (40 ×). (C) Score 1 (weak) in well-differentiated endometriosis histological type (40 ×).

The mean age was 36.1 ± 5.3 years, and the Body Mass Index (BMI) was 23.6 ± 4.8 kg/m^2^. All the endometriotic lesions expressed Ezrin in the glands, being 7 (12.3%) weak intensity, 30 (52.6%) moderate intensity, and 20 (35.1%) strong intensity (Fig. 1). Phosphorylated Ezrin was expressed in the stroma of all endometriotic lesions (Fig. 2), being 9 (15.8%) with weak intensity, 45 (78.9%) with moderate, and 3 (5.3%) with strong intensity (Table 1). There was no difference in the Ezrin and Phosphorylated Ezrin's expression in the retrocervical, ovarian, superficial, and intestinal lesions in the same patient.

All the three groups with different immunostaining intensities for Ezrin were similar in age, BMI, menstrual cycle phase, and endometriosis ASRM stage (Table 2). The authors observed that the symptoms’ prevalence such as dysmenorrhea, deep dyspareunia, acyclic pain, and dysuria were also similar between all the three different groups that were immunostaining positive for Ezrin. Although, when dyschezia was evaluated, the authors found an inversely proportional relationship between severe dyschezia (VAS ≥ 7) and Ezrin's intensity, being in patients with severe dyschezia 66.7% of Ezrin 1 (weak intensity), 36.7% of Ezrin 2 (moderate intensity), and 10.0% of Ezrin 3 (p = 0.013) (Table 2). There was no difference in Phospho-Ezrin expression and clinical characteristics, ASRM stage, and symptoms.

**Table 2:** Menstrual cycle phase, type of endometriotic lesion, stage of the disease, and Phospho-Ezrin immunostaining.

Characteristics	Phospho-Ezrin			p
	1	2	3	
Age (years)	39.4 ± 3.8	35.5 ± 5.4	35.0 ± 3.6	0.895^a^
BMI (kg/m^2^)	23.2 ± 2.3	23.8 ± 5.3	23.0 ± 1.0	
Phase of the menstrual cycle				
Proliferative	3 (33.3)	20 (44.4)	0 (0)	0.103^b^
Secretory	6 (66.7)	25 (55.6)	3 (100)	
ASRM Stage				0.828^c^
I	0 (0)	4 (8.9)	0 (0)	
II	5 (55.6)	14 (31.1)	1 (33.3)	
III	1 (11.1)	9 (20.0)	0 (0)	
IV	3 (33.3)	18 (40.0)	2 (66.7)	
Dysmenorrhea (VAS)				0.193^c^
< 7	4 (50.0)	10 (22.2)	1 (33.3)	
≥ 7	4 (50.0)	35 (77.8)	2 (66.7)	
Dyspareunia (VAS)				0.538^c^
< 7	7 (87.5)	30 (66.7)	2 (66.7)	
≥ 7	1 (12.5)	15 (33.3)	1 (33.3)	
Acyclic pain (VAS)				0.732^c^
< 7	6 (75.0)	30 (66.7)	3 (100)	
≥ 7	2 (25.0)	15 (33.3)	0 (0)	
Cyclic dyschezia (VAS)				0.633^c^
< 7	5 (62.5)	31 (68.9)	3 (100)	
≥ 7	3 (37.5)	14 (31.1)	0 (0)	
Cyclic dysuria (VAS)				0.100^c^
< 7	8 (100)	43 (95.6)	3 (100)	
≥ 7	0 (0)	2 (4.4)	0 (0)	
Infertility				0.822^c^
No	1 (11.8)	8 (22.9)	0 (0)	
Yes	8 (88.9)	27 (77.1)	3 (100)	

Data expressed as mean±standard deviation or n (%); BMI, Body Mass Index; ASRM, American Society for Reproductive Medicine, 1996; VAS, Visual Analogic Scale from 0 to 10.

a ANOVA.

b Kruskal-Wallis Test.

c Fischer's Exact Test.

The retrocervical lesions were of the stromal + well-differentiated glandular histological type in 4.7%, mixed in 71.4%, and undifferentiated in 23.8% of cases. There was no significant difference between the histological types of retrocervical lesions and Ezrin (p = 0.906) (Table 3) or phosphorylated Ezrin (p = 0.846) (Table 4) staining. The lesions of deep intestinal endometriosis were mostly stromal + mixed glandular (62.8%) histological type, followed by undifferentiated (20.0%), and well-differentiated (17.2%). There was no difference in Ezrin (p = 0.771) or phosphorylated Ezrin (p = 0.257) staining between the histological types of deep intestinal lesions. Superficial endometriosis lesions were stromal + well-differentiated glandular histological type in 2.8% of cases, mixed in 37.1%, and undifferentiated in 60.0%, with no difference in Ezrin (p = 0.077) or phosphorylated Ezrin (p = 0.919) expression. Ovarian endometriosis lesions were well-differentiated in 37.5% and undifferentiated in 62.5% of cases, also with no difference in Ezrin (p = 0.156) or Ezrin phosphorylated (p = 0.501) expression.

**Table 3:** Comparison of endometriotic lesions histological type and location to Ezrin immunostaining.

Histological type	Ezrin 1		Ezrin 2		Ezrin 3		p
	n	%	n	%	n	%	
**Deep retrocervical**							0.906^a^
Estromal pure	0	0.0%	1	100.0%	0	0.0%	
Glandular differentiated	1	50.0%	1	50.0%	0	0.0%	
Glandular mixed	4	13.3%	14	46.7%	12	40.0%	
Glandular undifferentiated	1	10.0%	5	50.0%	4	40.0%	
**Deep intestinal**							0.771^a^
Estromal pure	0	0	0	0	0	0	
Glandular differentiated	1	20.0%	2	40.0%	2	40.0%	
Glandular mixed	3	14.3%	13	61.9%	5	23.8%	
Glandular undifferentiated	0	0.0%	3	50.0%	3	50.0%	
**Superficial lesion**							0.077^a^
Estromal pure	0	0	0	0	0	0	
Glandular differentiated	1	100.0%	0	0.0%	0	0.0%	
Glandular mixed	3	23.1%	5	38.5%	5	38.5%	
Glandular undifferentiated	1	4.8%	10	47.6%	10	47.6%	
**Ovarian lesion**							0.156^a^
Estromal pure	0	0	0	0	0	0	
Glandular differentiated	1	33.3%	0	0.0%	2	66.7%	
Glandular mixed	0	0	0	0	0	0	
Glandular undifferentiated	1	20.0%	2	40.0%	2	40.0%	

a Fischer's exact test.

**Table 4:** Comparison of endometriotic lesions histological type and location to Phosphorylated Ezrin immunostaining.

Histological type	Phospho-Ezrin 1		Phospho-Ezrin 2		Phospho-Ezrin 3		p
	n	%	n	%	n	%	
**Deep retrocervical**							0.846^a^
Estromal pure	0	0.0	1	100.0	0	0.0	
Glandular differentiated	0	0.0	2	100.0	0	0.0	
Glandular mixed	4	13.3	24	80.0	2	6.7	
Glandular undifferentiated	2	20.0	7	70.0	1	10.0	
**Deep intestinal**							0.257^a^
Estromal pure	0	0.0	0	0.0	0	0.0	
Glandular differentiated	0	0.0	5	100.0	0	0.0	
Glandular mixed	5	23.8	13	61.9	3	14.3	
Glandular undifferentiated	0	0.0	6	100.0	0	0.0	
**Superficial lesion**							0.919^a^
Estromal pure	0	0.0	0	0.0	0	0.0	
Glandular differentiated	0	0.0	1	100.0	0	0.0	
Glandular mixed	2	15.4	10	76.9	1	7.7	
Glandular undifferentiated	4	19.0	17	81.0	0	0.0	
**Ovarian lesion**							0.501^a^
Estromal pure	0	0.0	0	0.0	0	0.0	
Glandular differentiated	0	0.0	3	100.0	0	0.0	
Glandular mixed	0	0.0	0	0.0	0	0.0	
Glandular undifferentiated	1	20.0	3	60.0	1	20.0	

a Fischer's Exact test.

## Discussion

Many factors have been related to endometriotic cells’ implantation in the pelvic cavity, such as a receptive environment that would allow the implantation and proliferation of ectopic endometriotic cells and the capacity to adhere to the peritoneal surface associated with an aberrant immune response.^1^^,^^8^ There are many studies in the literature reporting the importance of the Ezrin protein in the metastatic process and its interaction with the matrix extracellular remodeling during the malignant cells’ invasion in healthy adjacent tissues.^8^^,^^14^, ^15^, ^16^ Like in cancer, the Ezrin protein was also evaluated in some studies related to endometriosis’ invasiveness, increasing cell migration and adhesion in ectopic endometrial cells, mainly due to its capacity in remodeling and organizing the cytoskeleton.^17^^,^^18^ Nonetheless, its real role in the pathogenesis of endometriosis remains poorly investigated. In this study, the authors evaluated the possible role of Ezrin and Phospho-ezrin in different types of lesions of endometriosis.

The present study's results showed that regardless of the location, all endometriotic lesions had positive staining for Ezrin in the glands and for its activated form, Phospho- Ezrin, in the stroma, claiming that Ezrin activation may play an important role in the attachment of the endometrial cells on the peritoneal surface especially due to its ability in remodeling the cytoskeleton and cell-cell and cell-matrix adhesion.^17^ In the same way, in 2008, Ornek et al.^13^ reported that the stroma of ectopic endometrium of women with endometriosis had high-intensity levels of Ezrin and Phospho Ezrin when compared with women without endometriosis. Besides this, the stroma of endometriotic lesions had more pronounced invasive characteristics such as increased protrusions and pseudopodia, and very high-intensity staining of Phospho Ezrin when compared to the control group, suggesting that there might be an association between this protein and invasiveness.

In another study, Jiang et al.^18^ reported that after blocking Ezrin with small interfering RNA (siRNA) they observed a reduced migration of the ectopic endometrium cells associated with a decreased expression of GTPases and kinase protein associated with RhoA, RhoC and ROCK1.^8^^,^^18^ These Rho families are involved in the regulation of diverse cellular functions including cytoskeletal organization, growth, and differentiation.^17^^,^^18^ The expression of Rho/ROCK has also been described to be increased in some tumors, particularly during their progression to more invasive phenotypes.^18^^,^^19^ The overexpression of RhoA and RhoC can disrupt cell-cell junctions and induce the migration of tumor cells promoting the adhesions between cells and the extracellular matrix.^14^^,^^15^ Moreover, Moggio et al.^16^ observed a decrease in proliferation, motility, and phosphorylation of Ezrin and also a reduction of HIF-1α and VEGF expression in ectopic mesenchymal stem cells when compared to healthy endometrium, after using a potent immunomodulator, multi-tyrosine inhibitor called Sorafenib.

The present study observed an inversely proportional relationship between severe dyschezia and Ezrin's immunostaining intensity, whereas there was no significant difference in relation to other symptoms. Intestinal endometriotic lesions have intense fibrosis, and this may be associated with the repeated process of tissue injury and remodeling leading to a mechanism called Epithelial-Mesenchymal Transition (EMT), responsible for the temporary loss of mesothelial barrier integrity.^7^ In the absence of this barrier, the endometrial cells are more capable in adhering to the peritoneal stroma and establishing the endometriotic lesions, increasing the intensity of the symptoms.^20^ Evidence suggests that Ezrin is important in EMT by regulating and activating NF-κB, which has a critical role in promoting tumor progression and invasion.^21^ Previous studies with cancer patients also have shown that the Ezrin, Radixin, and Moesin complex interferes in the expression of membrane receptors, opioid metabolization, and pain modulation, with its activated form being related to greater resistance to the effect of opioids and greater presence of central sensitization, while the inactive form would be related to the opposite effect, similar to what the authors have observed in dyschezia.^22^^,^^23^

Ezrin is an important protein in the cycling endometrium and is in part regulated by estrogen, the main hormone of the proliferative phase. Hormonal effects in the endometrium during the menstrual cycle are mirrored by cytologic changes regulated by the actin cytoskeleton, and Phospho-Ezrin is required for cytoskeleton rearrangements and for the mechanism of cell-cell attachment.^24^ Even being known that Ezrin expression responds to estrogen, the authors did not find any significant difference between Ezrin and Phospho Ezrin proteins according to the menstrual cycle phase. However, Tan et al. reported the presence of these proteins in the secretory phase, at the peak of estrogen and progesterone, and the gravitation of them in the periphery of secretory vacuoles, microvilli, and pinopodes.^25^ These last structures are related to the implantation and adhesion during the luteal phase, and to important markers of endometrial receptivity. Over more, Ezrin can interact with thrombomodulin, a membrane protein that has an important role in placentation. This complex Ezrin-Thrombomodulin promotes interaction with the actin filaments of the cytoskeleton and consequently the expression and organization of pinopodes.^26^

## Conclusion

The authors observed the expression of both Ezrin protein and its activated form Phospho Ezrin in all the endometriotic lesions, providing great evidence that these proteins can be considered important markers to explain the migration and attachment of endometriotic lesions. At this point, it is still unclear if Ezrin and Phospho-Ezrin are a cause or consequence of endometriosis. Further studies comparing different types of lesions and eutopic endometrium are necessary to elucidate the real role of these proteins in the pathogenesis of endometriosis.

## Authors' contributions

MPA and MAS recruited the participants and followed them up. All authors contributed equally to designing the study, writing the manuscript, and final approval of the version.

## Conflicts of interest

The authors declare no conflicts of interest.
